# Lenalidomide increases human dendritic cell maturation in multiple myeloma patients targeting monocyte differentiation and modulating mesenchymal stromal cell inhibitory properties

**DOI:** 10.18632/oncotarget.18085

**Published:** 2017-05-23

**Authors:** Federica Costa, Rosanna Vescovini, Marina Bolzoni, Valentina Marchica, Paola Storti, Denise Toscani, Fabrizio Accardi, Laura Notarfranchi, Benedetta Dalla Palma, Cristina Manferdini, Sabrina Manni, Giannalisa Todaro, Gina Lisignoli, Francesco Piazza, Franco Aversa, Nicola Giuliani

**Affiliations:** ^1^ Department of Medicine and Surgery, University of Parma, Parma, Italy; ^2^ Hematology and BMT Center, Azienda Ospedaliero-Universitaria di Parma, Parma, Italy; ^3^ Laboratory of Immunorheumatology and Tissue Regeneration, Istituto Ortopedico Rizzoli, Bologna, Italy; ^4^ Department of Medicine, Hematology Branch, University of Padova and Venetian Institute of Molecular Medicine, Padova, Italy

**Keywords:** multiple myeloma, dendritic cells, lenalidomide, mesenchymal stromal cells, immunomodulation

## Abstract

The use of Lenalidomide (LEN), to reverse tumor-mediated immune suppression and amplify multiple myeloma-specific immunity is currently being explored. Particularly, LEN effects on dendritic cells (DCs) are still unclear. In this study, we investigated the potential effect of LEN on DC differentiation and activity. DCs were differentiated either from CD14^+^ cells obtained from patients with multiple myeloma or from a human monocytic cell line.

LEN, at the concentration range reached *in vivo*, significantly increased the median intensity expression of HLA-DR, CD86 and CD209 by DCs derived from both bone marrow and peripheral myeloma monocytes and enhanced the production of Interleukin-8, C-C motif chemokine ligand (CCL) 2, CCL5 and tumor necrosis factor-α. Consistently, LEN pre-treated DCs showed an increased ability to stimulate autologous CD3^+^ cell proliferation. LEN effect on dendritic differentiation was associated with the degradation of the Cereblon-related factors Ikaros and Aiolos. Moreover, we showed that LEN also blunted mesenchymal stromal cell inhibitory effect on dendritic differentiation, inhibiting Casein Kinase-1α levels. Finally, *in vitro* data were confirmed in *ex vivo* cultures obtained from relapsed myeloma patients treated with LEN, showing a significant increase of DC differentiation from peripheral blood monocytes.

In conclusion, LEN increased the expression of mature dendritic markers both directly and indirectly and enhanced DC ability to stimulate T cell proliferation and to release chemokines. This suggests a new possible mechanism by which LEN could exert its anti-myeloma activity.

## INTRODUCTION

The Immunomodulatory drugs (IMiDs^®^) are a group of therapeutic agents, Thalidomide-derivatives, including Lenalidomide (LEN) and Pomalidomide (POM). The development of these drugs represented a paradigm shift in the treatment of multiple myeloma (MM) [1, 2]. LEN-based regimen is one of the standard of care for MM patients either in frontline or in relapsed setting [3–5]. Moreover, LEN has shown a significant impact on the progression free survival in maintenance after autologous stem cell transplantation [6].

Several mechanisms of action have been described, [7, 8] including immunomodulatory effects as T helper 1 cell activation, T regulatory cell (T reg) suppression, and the induction of antibody-dependent cellular cytotoxicity (ADCC) by natural killer (NK) cells [9]. The molecular mechanisms involved in the anti-MM effect of IMiDs^®^ have been recently elucidated highlighting the role of Cereblon (CRBN) and its target factors [10, 11]. LEN binds CRBN in MM cells and causes selective ubiquitination and degradation, by the CRBN-Cullin-RING E3 ubiquitin ligase (CRL) 4 complex, of two lymphoid transcription factors essential for MM survival, Ikaros and Aiolos [12]. Through the same mechanism, LEN enhances T cell proliferation and interleukin (IL)-2 production. The loss of Ikaros and Aiolos is necessary and sufficient for LEN therapeutic effect [13]. Recently, it has been also reported a distinct CRBN substrate, Casein Kinase 1 alpha (CK1-α) that is ubiquitinated and degraded after LEN treatment in myelodysplastic syndrome (MDS) with deletion of chromosome 5q (del(5q)) [14] and in MM, as reported by Manni S et al. [15].

Currently, few data are reported on the possible effects of LEN on dendritic cells (DCs) populations. [9, 16–18] Interestingly, it has been shown that DCs from peripheral blood (PB) were functionally defective in MM patients, since they had decreased expression of maturation markers and antigen presentation capacity [19, 20]. Nevertheless, it is not known which is the role of DCs in the anti-MM effect of LEN.

Different studies reported an increased incidence of acute Graft versus host disease (aGvHD), with a possible enhancement of the graft versus MM effect, in patients treated with LEN after allo-transplantation [21–23]. Since it is known that host and donor DCs are critical in the development of GvHD and also involved in the immunosuppressive properties of mesenchymal stromal cells (MSCs); [24–26] it is conceivable that LEN affects DCs.

Based on these evidences, in this study we investigated whether LEN may affect maturation, phenotype and functionality of DCs as antigen presenting cells (APCs), either directly or through the modulation of human mesenchymal stromal cell (hMSC) effect on DCs.

## RESULTS

### LEN enhanced *in vitro* DC differentiation from both bone marrow (BM) and PB monocytes of MM patients and increased their chemokine and cytokine production degrading Ikaros and Aiolos

**Figure 1:**
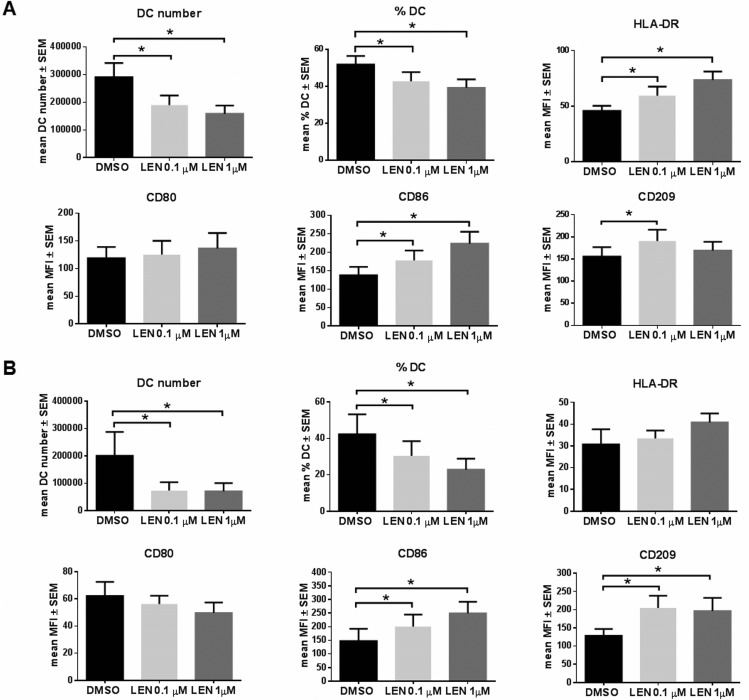
LEN enhanced *in vitro* DC differentiation from both BM and PB of MM patients (**A**) DCs were differentiated from BM CD14^+^ cells of MM patients, cultured in RPMI 10% FBS with IL-4 and GM-CSF, for 8 days, in presence of LEN (0.1 and 1 μM) or DMSO. TNF-α was added in the last 24 h of differentiation period. Non-adherent cells were collected and analysed by flow-cytometry for DC maturation markers. Graph bars represent the mean of DC number and % and median fluorescent intensity (MFI) of DC maturation markers ± standard error of the mean (SEM) (*p* calculated by paired Student's *t*-test) of 19 independent experiment. (**B**) DCs were differentiated from PB of 6 MM patients, following the same protocol. Graph bars represent the mean of DC number and % and MFI of DC maturation markers ± SEM (*p* calculated by paired Student's *t*-test).

We analyzed the expression of DC maturation markers on monocytes derived-DCs (mo-DCs) differentiated from BM aspirates and PB of MM patients. Despite a reduction of both number and % of mature DCs, LEN, at the concentration range reached *in vivo* in MM patients, [27] significantly increased the expression, by DCs derived from BM, of HLA-DR (mean median fluorescence intensity (MFI) ± standard error of the mean (SEM): DMSO *vs* LEN 0.1 μM, 45.82 ± 4.55 *vs* 59.45 ± 8.21, *p* = 0.029; DMSO *vs* LEN 1 μM, 45.82 ± 4.55 *vs* 73.52 ± 7.71, *p* = 0.001), CD86 (mean MFI ± SEM: DMSO *vs* LEN 0.1 μM, 137.58 ± 22.83 *vs* 177.76 ± 27.04, *p* = 0.036; DMSO *vs* LEN 1 μM, 137.58 ± 22.83 *vs* 223.38 ± 32.26, *p* = 0.003) and CD209 (mean MFI ± SEM: DMSO *vs* LEN 0.1 μM, 155.80 ± 21.06 *vs* 190.73 ± 25.35, *p* = 0.004) (*p* calculated by paired Student's *t*-test) (Figure 1A), compared to vehicle (DMSO). Flow-cytometry histograms from one representative MM patient were reported in Supplementary Figure 1A.

Similarly to BM derived DCs, increased CD86 (mean MFI ± SEM: DMSO *vs* LEN 0.1 μM, 147.49 ± 45.08 *vs* 200.44 ± 44.22, *p* = 0.002; DMSO *vs* LEN 1 μM, 147.49 ± 45.08 *vs* 249.61 ± 42.10, *p* = 0.016) and CD209 (mean MFI ± SEM: DMSO *vs* LEN 0.1 μM, 128.69 ± 18.09 *vs* 204.88 ± 33.54, *p* = 0.008; DMSO *vs* LEN 1 μM, 128.69 ± 18.09 *vs* 196.32 ± 36.33, *p* = 0.023) (*p* calculated by paired Student's *t*-test) expression was found in DCs differentiated from PB CD14^+^ cells (Figure 1B). Flow-cytometry histograms from one representative patient were reported in Supplementary Figure 1B. Any significant differences between BM and PB samples of the same patient were found on LEN effect on DC maturation markers (2way ANOVA) (data not shown).

**Figure 2:**
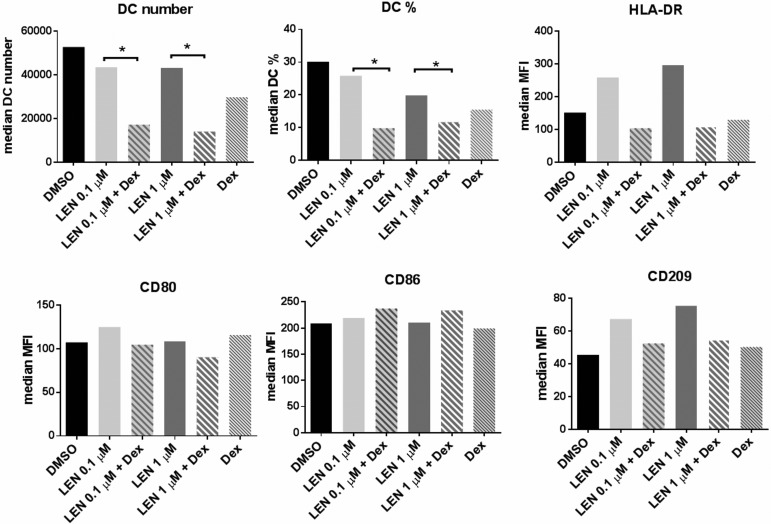
LEN effects on DC maturation markers were abrogated by Dex DCs were differentiated from BM CD14^+^ cells of MM patients, in the presence of LEN (0.1 and 1 μM) or DMSO, as reported in Materials and Methods. At the end of culture period, cells were collected and reseeded (5 × 10^4^/ml) in fresh medium with Dex (10^−8^ M) or vehicle (EtOH) for 48 h. After Dex treatment, cells were collected and analyzed for DC maturation markers. Graph bars represent the median of DC number and % and MFI of DC maturation markers from 4 independent experiments (*p* calculated by Wilcoxon test).

Interestingly, the increased expression of DC maturation markers was abrogated when LEN was used in combination with Dexamethasone (Dex) at 10^−8^ M (Dex *vs* LEN 0.1 μM + Dex *vs* LEN 1 μM + Dex median MFI, HLA-DR: 129.5 *vs* 103.9 vs 109.9; CD86: 199 *vs* 237.4 *vs* 233.5; CD80: 115 *vs* 104.6 *vs* 90.24; CD209: 50.28 *vs* 52.58 *vs* 54.91, no statistically significant differences) (Friedman test) (Figure 2). Moreover, the addition of Dex dramatically decreased the number of DCs (median number: LEN 0.1 μM + Dex *vs* LEN 0.1 μM, 17238 *vs* 43568, *p* < 0.05; LEN 1 μM + Dex *vs* LEN 1 μM, 14028 *vs* 43283, *p* < 0.05) and the percentage of DCs obtained *in vitro* (median DC%: LEN 0.1 μM + Dex *vs* LEN 0.1 μM, 9.85 *vs* 25.90, *p* < 0.05; LEN 1 μM + Dex *vs* LEN 1 μM, 11.69 *vs* 19.9, *p* < 0.05) (*p* calculated by Wilcoxon test) (Figure 2).

**Figure 3:**
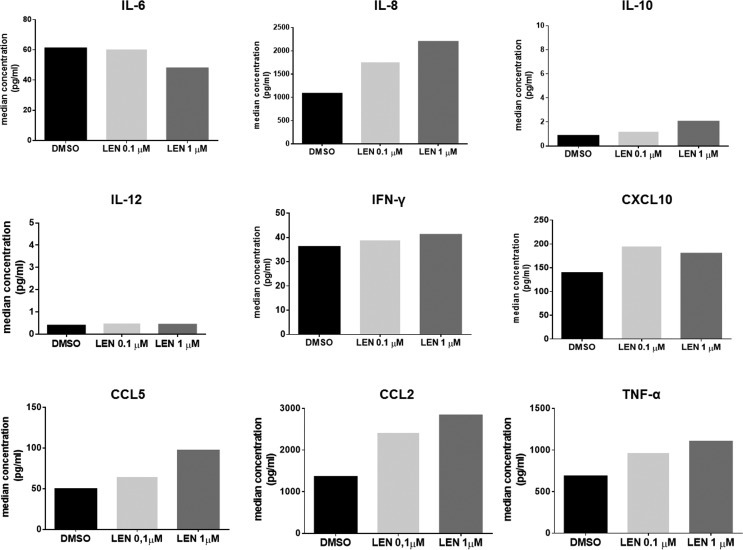
LEN increased the production of IL-8, CCL2, CCL5 and TNF-α by DCs DC CM was collected after *in vitro* DC differentiation from BM CD14^+^ cells of 5 MM patients. The levels (pg/ml) of cytokines and chemokines involved in immune response were evaluated by a Bio-Plex^®^ Multiplex System. Graph bars represent the median concentration of soluble factor levels in the presence of DMSO or LEN. For TNF-α level evaluation, the obtained results were normalized for TNF-α concentration measured in the control medium (RPMI 10% FBS, with IL-4, GM-CSF and TNF-α at concentration used during DC differentiation).

Then we investigated the potential effect of LEN treatment on cytokine production by BM DCs using a Multiplex ELISA assay. We found that LEN treatment enhanced the production of IL-8 (median concentration, DMSO *vs* LEN 0.1 μM *vs* LEN 1 μM: 1076 *vs* 1755 *vs* 2193 pg/ml, *p* < 0.05), CC chemokine ligand (CCL)2 (median concentration, DMSO *vs* LEN 0.1 μM *vs* LEN 1 μM: 1355 *vs* 2414 *vs* 2831 pg/ml, *p* < 0.05), CCL5 (median concentration, DMSO *vs* LEN 0.1 μM *vs* LEN 1 μM: 49.68 *vs* 64.48 *vs* 96.94 pg/ml, *p* < 0.05) and TNF-α (median concentration, DMSO *vs* LEN 0.1 μM *vs* LEN 1 μM: 684.4 *vs* 965.4 *vs* 1101 pg/ml, *p* < 0.05) and slightly decreased the production of IL-6 (median concentration, DMSO *vs* LEN 0.1 μM *vs* LEN 1 μM: 60.97 *vs* 60.30 *vs* 47.74 pg/ml, *p* < 0.05) (*p* calculated by Friedman test), by mo-DCs differentiated from MM patients, compared to vehicle (Figure 3).

**Figure 4:**
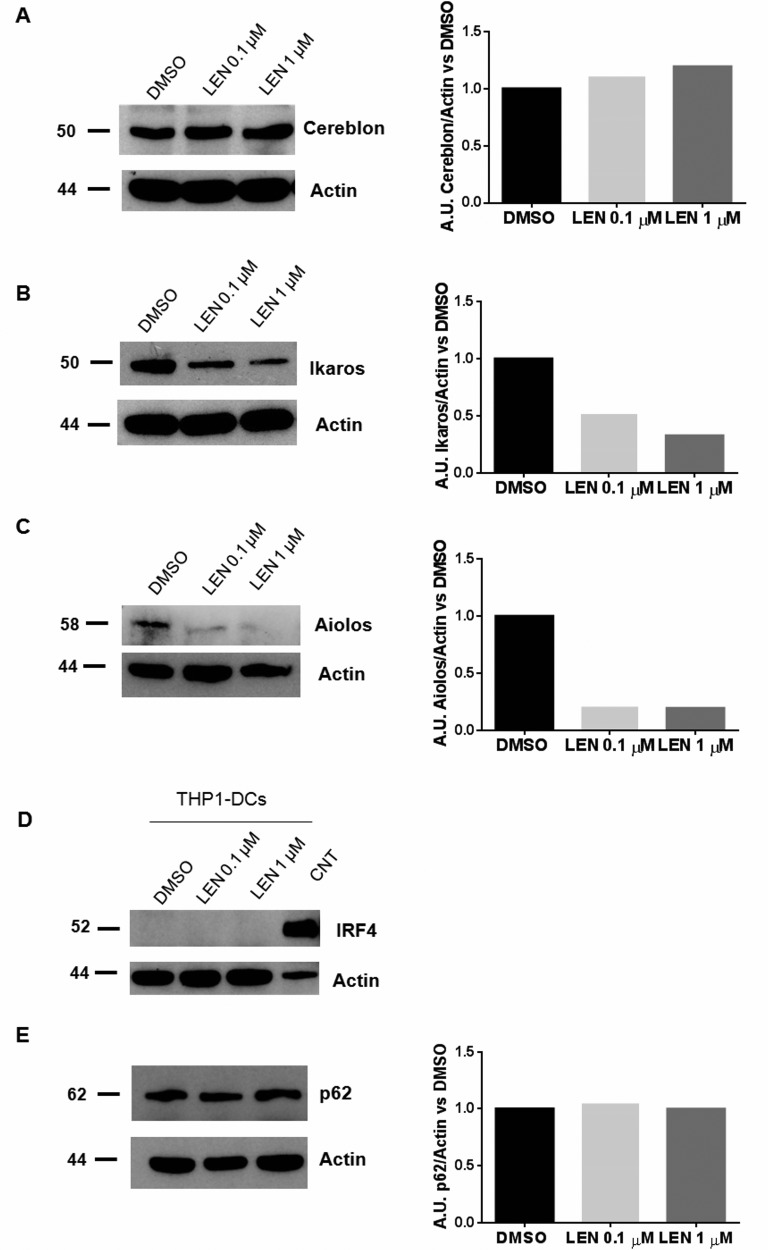
LEN effect on DC differentiation was mediated by Ikaros and Aiolos degradation DCs were differentiated from THP-1 cell line, by adding rhIL-4 (200 ng/ml), rhGM-CSF (100 ng/ml), ionomycin (200 ng/ml) and rhTNF-α (20 ng/ml) for 72 h to the culture medium (RPMI 1664, serum depleted); then LEN (0.1 and 1 μM) or DMSO were added for the last 24 h of culture period. Differentiated cells (THP1-DCs) were then collected and Cereblon (**A**), Ikaros (**B**), Aiolos (**C**), IRF4 (**D**) and p62 (**E**) protein levels were analyzed by Western Blotting. β-actin was used as internal control.

Based on the literature data showing that LEN exerts the anti-MM activity through the selective ubiquitination and degradation of Cereblon targets, Ikaros and Aiolos [12], we assessed the protein levels of Cereblon, Ikaros and Aiolos on LEN-treated THP1-DCs. We showed that THP1-DCs expressed Cereblon (Figure 4A) and that LEN treatment down-regulated Ikaros protein levels in a dose-dependent manner (Figure 4B). Moreover, we found that the basal Aiolos protein level was very low in THP1-DCs and LEN further decreased its expression in these cells (Figure 4C). We also tested the effect on two other Cereblon targets, Interferon regulatory factor (IRF) 4 and Sequestosome-1/SQSTM1 (p62), that were respectively down-regulated [28] and up-regulated [29] after LEN treatment in MM cells. We showed that THP1-DCs did not express IRF4 (Figure 4D) and LEN did not affect p62 in THP-1 DCs (Figure 4E).

### LEN enhanced DC ability to stimulate autologous CD3^+^ cell proliferation

Next we evaluated whether the effect of LEN on DC maturation may affect DC functional properties. In order to evaluate LEN effect on DC ability to stimulate T cell proliferation, DCs differentiated from BM of 4 MM patients, were tested as stimulators in the autologous Mixed Lymphocyte Reaction (MLR) assay in the presence of LEN or vehicle.

**Figure 5:**
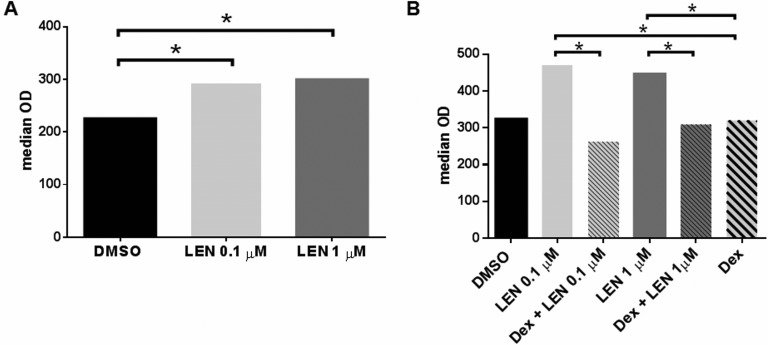
Effect of LEN on DC ability to stimulate T cell proliferation, alone and in combination with Dex DCs were differentiated from BM CD14^+^ cells of 6 MM patients, in the presence of LEN or DMSO, alone (**A**) or in combination with Dex (**B**), as previously reported. At the end of culture period, cells were collected and reseeded (3 × 10^3^ cells/w) in round-bottomed 96well-plates, in RPMI 1640 with 15% AB human serum. DCs were co-cultured with autologous PB CD3^+^ cells (1 × 10^4^/w) for 6 days. At the end of culture period, an MTT assay was performed in order to measure T cell proliferation. Graph bars represent the median O.D. of T cell proliferation in co-culture with pre-treated DCs (*p* calculated by Mann-Whitney test).

Interestingly, CD3^+^ cell proliferation was significantly higher in co-culture with LEN-treated DCs, compared to DMSO-treated DCs (median OD: DMSO *vs* LEN 0.1 μM, 225 *vs* 292, *p* = 0.0279; DMSO *vs* LEN 1 μM, 225 *vs* 299, *p* = 0.0045) (*p* calculated by Mann-Whitney test) (Figure 5A). However, this effect was abrogated in the presence of Dex at 10^−8^ M (median OD: LEN 0.1 μM + Dex *vs* LEN 0.1 μM, 263 *vs* 470, *p* = 0.0043; LEN 0.1 μM *vs* Dex, 470 *vs* 320, *p* = 0.0043; LEN 1 μM + Dex *vs* LEN 1 μM, 309 *vs* 446.5, *p* = 0.0317; LEN 1 μM *vs* Dex, 446.5 *vs* 320, *p* = 0.0159) (*p* calculated by Mann-Whitney test) (Figure 5B).

### LEN blunted hMSC inhibitory properties on DC differentiation inhibiting CK1-α levels

To investigate a possible MSC-mediated indirect effect of LEN on DCs, BM CD14^+^ cells of MM patients were differentiated into DCs in the presence of LEN or DMSO treated human telomerase reverse transcriptase transduced hMSC (hTERT-hMSC) conditioned medium (CM).

**Figure 6:**
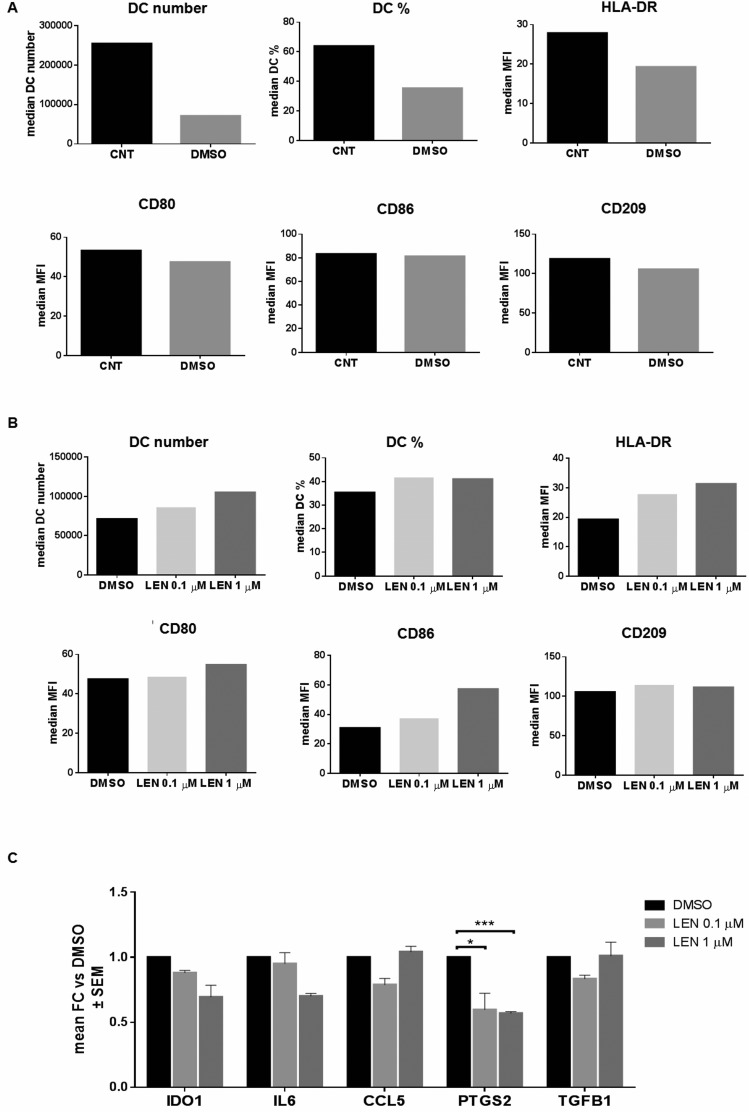
LEN blunted hTERT-hMSC inhibitory properties on DC differentiation through the down-regulation of PTGS2 expression levels DCs were differentiated from BM CD14^+^ cells of 3 MM patients, in the presence or absence of the CM (ratio 1:2 with RPMI 1640 10% FBS, with IL-4 and GM-CSF) of hTERT-hMSCs, treated with LEN (0.1 and 1 μM) or DMSO for 5 days. At the end of culture period, cells were collected and analyzed for DC maturation markers, by flow-cytometry. (**A**) The inhibitory effect of hTERT-hMSCs on *in vitro* DC differentiation was firstly checked. Graph bars represent the median of DC number and % and MFI of DC maturation markers on DCs cultured with DMSO-treated hTERT-hMSCs CM or CNT (RPMI 10% FBS). (**B**) The effect of LEN treated hTERT-hMSCs on DC differentiation was then evaluated. Graph bars represent the median of DC number and % and MFI of DC maturation markers on DCs cultured with LEN-treated hTERT-hMSCs CM or DMSO. (**C**) hTERT-hMSCs were seeded in T75 flasks and cultured in RPMI 10% FBS, in presence of LEN (0.1 and 1 μM) or DMSO, for 5 days. At the end of culture period, cell pellets were collected and analyzed by RT-PCR for the mRNA expression of several immunosuppressive factors. Graph bars represent the mean Fold Change (FC) of LEN treated hTERT-hMSCs *vs* DMSO ± SEM of 3 independent experiments (*p* calculated by Student's *t*-test; *p < 0.05, ***p < 0.0001).

We firstly demonstrated that hTERT-hMSC CM decreased DC maturation marker expression in this *in vitro* system (Figure 6A); then we found that LEN treatment reverted this effect, by increasing HLA-DR (median MFI, DMSO *vs* LEN 0.1 μM *vs* LEN 1 μM: 19.88 *vs* 27.88 *vs* 31.34, *p* < 0.05) and CD86 (median MFI, DMSO *vs* LEN 0.1 μM *vs* LEN 1 μM: 30.78 *vs* 37.52 *vs* 57.25, *p* < 0.05) (*p* calculated by Friedman test) (Figure 6B).

Thereafter, we examined whether LEN treatment affected the expression of immunosuppressive factors in hTERT-hMSCs, by Real-time PCR (RT-PCR). Interestingly, we found that LEN significantly down-regulated prostaglandine 2 (*PTGS2*) gene expression levels at all tested concentrations (LEN 0.1 μM *vs* DMSO, *p* = 0.0326; LEN 1 μM *vs* DMSO, *p* < 0.0001) (Figure 6C) but not indoleamine 2,3-dioxygenase 1 (*IDO1*), *IL6*, *CCL5* and transforming growth factor beta 1 (*TGFB1*).

**Figure 7:**
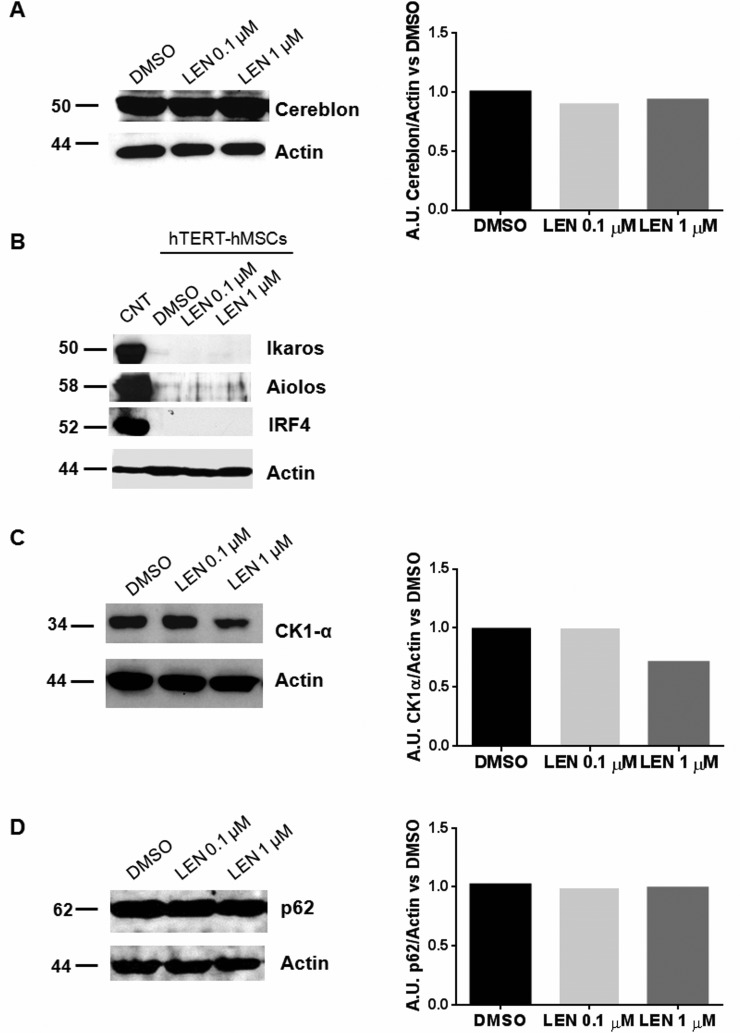
LEN treatment decreased CK1-α levels in hTERT-hMSCs hTERT-hMSCs were seeded in T75 flasks and cultured in RPMI 10% FBS, in presence of LEN (0.1 and 1 μM) or DMSO, for 5 days. At the end of culture period, cell pellets were collected and analyzed by Western Blotting for Cereblon (**A**), Ikaros (**B**), Aiolos (B), IRF4 (B), CK1-α (**C**) and p62 (**D**). β-actin was used as internal control.

To investigate the molecular mechanism involved in the effect of LEN on MSCs, firstly we checked the expression profile of Cereblon and its target proteins in hTERT-hMSCs showing that they expressed Cereblon (Figure 7A) but not Ikaros, Aiolos and IRF4 (Figure 7B). We then focused our attention on another Cereblon substrate, CK1-α. [14] LEN, at the higher concentration, decreased CK1-α protein level in hTERT-hMSCs (Figure 7C). We also found that LEN treatment did not affect p62 protein level, as observed in THP1- DCs (Figure 7D).

To better clarify the correlation between the decreased CK1-α levels in hMSCs after LEN treatment and LEN effect on the immunosuppressive properties of MSCs on DCs, we down-regulated CK1α in hTERT-hMSCs through an IPTG inducible shRNA. We firstly checked CK1-α down-regulation by western blotting (Supplementary Figure 3A) and then we used the CM for *in vitro* DC differentiation from BM CD14^+^ cells of 2 MM patients. Interestingly, we found that the effect of hTERT-hMSCs on DC maturation markers were reverted by the down-regulation of CK1-α (mean MFI ± SEM: CNT *vs* wt hTERT-hMSCs *vs* hTERT-hMSCs 6044, HLA-DR: 108.8 ± 34.49 *vs* 76.74 ± 14.66 *vs* 106.7 ± 51.49; CD86: 103.7 ± 2.8 *vs* 111.9 ± 2.5 *vs* 177 ± 15.87; CD80: 60.22 ± 10.19 *vs* 40.39 ± 4.52 *vs* 100.9 ± 0.91; CD209: 47.90 ± 6.84 *vs* 40.22 ± 5.92 *vs* 74.75 ± 20.01) (Supplementary Figure 3B).

### LEN treatment of MM patients increased *ex vivo* DC differentiation

Finally, to evaluate the effect of *in vivo* LEN treatment on DC maturation markers, we compared the expression profile of DCs differentiated from PB CD14^+^ cells of 9 MM relapsed patients, purified at the baseline (DAY 0) and after 7 days of LEN treatment, just before the start of the weekly treatment with Dex. All the patients were responsive to LEN treatment.

**Figure 8:**
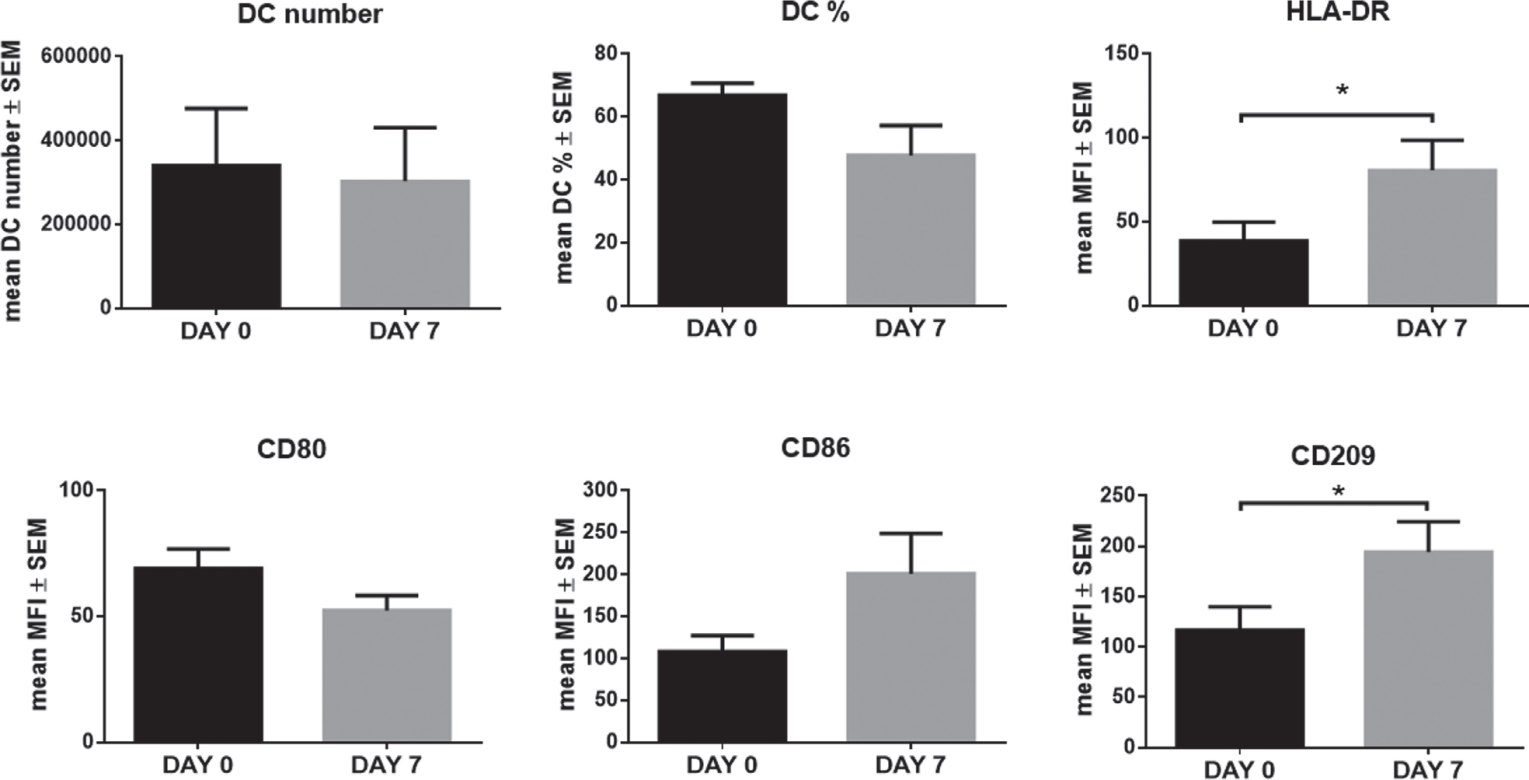
*In vivo* LEN treatment increased *in vitro* DC differentiation from CD14^+^ cells of MM patients DCs were *in vitro* differentiated from PB CD14^+^ cells of 9 MM patients at DAY 0 and after one week (DAY 7) of LEN 25mg/day treatment. Cells were cultured in RPMI 10% FBS with IL-4 and GM-CSF for 8 days and TNF-α was added for the last 24 h. At the end of culture period, cells were collected and analyzed for DC maturation markers, by flow-cytometry. Graph bars represent the mean of DC number and % and MFI of DC maturation markers at DAY 7 *vs* DAY 0 ± SEM, of 9 independent experiments (*p* calculated by paired Student's *t*-test).

Interestingly, we found that *in vivo* LEN treatment significantly increased the expression of HLA-DR (mean MFI ± SEM: DAY 7 *vs* DAY 0, 80.59 ± 11.21 *vs* 38.56 ± 17.99, *p* = 0.036) and CD209 (mean MFI ± SEM: DAY 7 *vs* DAY 0, 194.24 ± 30.22 *vs* 116.47 ± 23.47, *p* = 0.012), and CD86 without reaching statistical significance (mean MFI ± SEM: DAY 7 *vs* DAY 0, 200.75 ± 48.20 *vs* 107.95 ± 19.21, *p* = 0.075) (*p* calculated by paired Student's *t*-test) (Figure 8), as we showed for the *in vitro* treatment. The effect on CD209 expression was also observed after 21 days of LEN treatment (data not shown). Flow-cytometry histograms from one representative MM patient were reported in Supplementary Figure 4.

## DISCUSSION

DCs of MM patients are known to be functionally defective, with a decreased expression of maturation markers and antigen presentation ability [19]. The production by MM cells of several soluble factors with immunosuppressive properties on DCs, such as IL-6, IL-10, vascular endothelial growth factor and TGF-β, contributes to these immunological defects [19, 30, 31]. LEN exerts its immunomodulatory activity through several mechanisms of action on T and NK cells; [7, 32] however, few literature data reported the effect of LEN on DCs in the contest of the anti-MM activity and in the increased incidence of GvHD observed in patients treated with this drug [33].

Studies performed on murine models, showed that both LEN and POM treatment increased the expression of DC maturation markers, enhanced DC endocytotic activity, increased the production of TNF-α and CCL2, and the DC-dependent T-cell expansion [16, 17]. However, LEN effect on human DCs are still unclear. One study, recently published [18], reported that LEN (5 μg/ml ≈ 20 μM) enhanced the maturation of DCs generated from PB of MM patients and increased the ability to stimulate T cell proliferation in line with our results [18]. Particularly we found that LEN, at concentrations reached *in vivo* in MM patients treated with this drug 5–25 mg daily, [27, 34] significantly increased the expression of DC maturation markers, HLA-DR, CD86 and CD209, that are involved in DC co-stimulatory function and trafficking, [35–37] both in BM and PB of MM patients. This effect was abrogated in the presence of Dex at 10^−8^ M, consistent with several studies that reported Dex inhibitory effects on DC maturation and functions [38, 39]. However, contradictory data were reported on the antagonistic effects of Dex on LEN immunomodulatory properties. A study from Paiva et al. [40] on high-risk Smoldering MM (SMM) patients reported no significant differences in the immune profile of patients treated with LEN/Dex *vs* LEN alone [40]. Conversely, different studies showed a profound inhibition of LEN-mediated NK and T cell activation with Dex combination [41, 42].

Consistent with the effect of LEN on DC maturation markers, we found that LEN treatment increased DC production of IL-8, CCL2, CCL5, and TNF-α, in line with data observed on murine models [16, 17]. These factors regulate the antigen uptake and the activation of the innate immune system, and are chemo-attractants for various immune cells, [43] suggesting that LEN treatment enhanced the role of DC in linking innate and adaptive anti-tumor-antigen immune responses. In line with this hypothesis, we showed that LEN effect on *in vitro* DC differentiation was associated with an increased DC functional activity to stimulate T cell proliferation that was abrogated by the combination with Dex.

Subsequently, we investigated the molecular mechanism beyond LEN effects on DCs. It is widely demonstrated that LEN exerts its anti-MM activity through the modulation of Ikaros and Aiolos [12, 13]. Moreover, Ikaros deficiency in host APCs failed to enhance GvLeukemia despite increased GvHD severity, in a murine model of allo-transplantation [44]. Similarly, we found that both Ikaros and Aiolos were degraded in DCs after LEN treatment, as previously showed for MM cells and T cells [12, 13] [21].

Along with a direct effect of LEN on DC maturation, our data suggested a potential indirect effect, through the modulation of MSC immunomodulatory properties such as the production of cytokines and chemokines. [24–26, 45] When DCs were cultured with LEN-treated MSC CM, we observed an increased expression of DC maturation markers suggesting the ability of LEN to blunt the inhibitory effect of MSCs on DC differentiation. Moreover, we found that the treatment of MSCs with LEN significantly decreased the expression levels of *PTGS2*, known to inhibit the transitional processes of differentiation of monocytes into DCs [46, 47]. The molecular mechanisms involved in the effect of LEN on MSC was also investigated. Surprisingly, we lacked to find the expression of the main Cereblon targets, Ikaros and Aiolos, by MSCs but we showed that LEN caused the degradation of a novel described Cereblon substrate, CK1-α in MSCs. CK1-α is a component of the beta (β)-catenin destruction complex and a negative regulator of p53, and several studies [15, 48] recently reported that reduced CK1-α levels decrease MM cell survival and inhibit cell cycle progression [15, 48]. Interestingly, this factor seems to be involved in the resistance of plasma cells to LEN after long-term exposure [49].

Lastly, our *in vitro* evidences were expanded and confirmed by *ex vivo* DC cultures in relapsed MM patients treated with LEN 25 mg/day, as mono-therapy for one week, just before the start of the weekly treatment with Dex. After 7 days of treatment we found an increased PB DC differentiation. Of note, all analyzed patients were responsive to LEN treatment. This early effect was in agreement with recent data reporting the *in vivo* increase of T and NK cells, with a rapid decline of Ikaros, after 7 days of POM treatment without Dex in MM patients [50].

In conclusion, our data indicate that LEN increases the expression of mature DC markers both *in vitro* and in *ex vivo* cultures, enhancing DC ability to stimulate T cell proliferation and to release chemokines involved in the immune response. LEN treatment also reduces the immunosuppressive properties of hMSCs, suggesting new possible effects of IMiDs^®^ on the allo-reactivity against MM cells.

## MATERIALS AND METHODS

### Patients

BM and/or PB were obtained from 30 consecutive patients with active MM (50% female, 50% male; median age: 71 years, range 43–94), including both newly diagnosed and relapsed MM, admitted to our hematological Unit. Patient samples were obtained after informed consent, according to the Declaration of Helsinki. The study was approved by the Institutional Ethical Review Board of our Hospital.

Moreover, PB were obtained from 9 patients with relapsed MM (4 female, 5 male; median age: 73 years, range: 56–82; International Staging System (ISS) I: 4, International Staging System (ISS) II: 3, International Staging System (ISS) III: 2), at the baseline and after 7 days of treatment with LEN 25 mg/day (days 1–21), just before the start of the weekly treatment with Dex.

Mononuclear cells (MNCs) were isolated from BM and PB samples after Ficoll gradient separation and used for further *in vitro* studies.

### Cells and cell culture conditions

#### Cell lines

The human myeloma cell line (HMCL) JJN3, purchased by DSMZ (Braunschweig, Germany) and the human monocytic cell line THP-1, obtained from the American Type Culture Collection (Rockville, MD), were maintained in culture in RPMI 1640 medium with 10% FBS; hTERT-hMSCs were kindly gifted from Dr Giuseppe Gaipa (Monza, Italy) and maintained in culture with RPMI 10% FBS with hydrocortisone (10^−6^M). All cell lines were authenticated and tested for mycoplasma contamination.

#### Cell purification

BM and PB CD14^+^ cells were purified from total MNCs by an immuno-magnetic method using anti-CD14 mAb coated microbeads (MACS, Miltenyi Biotec; Bergisch-Gladbach, Germany). CD3^+^ cells were isolated following the same protocol, using anti-CD3 mAb from PB of MM patients. The presence of potential contaminating cells in each fraction was evaluated by flow cytometry analysis, using the fluorescence-activated flow cytometer BD FACS Canto II with Diva software (Becton, Dickinson and Company (BD); Franklin Lakes, NJ). Purity of cell samples was > 92%.

#### DC differentiation and cell treatment

DCs were differentiated from purified CD14^+^ cells, cultured *in vitro* at 1 × 10^6^ cells/ml in RPMI 10% FBS, with recombinant human (rh) granulocyte macrophage colony-stimulating factor (GM-CSF) (50 ng/ml) and IL-4 (50 ng/ml) (all purchased by Peprotech, Rocky Hill, NJ), for 8 days (replacing half media with fresh cytokines every 2–3 days), in the presence of LEN (purchased by Celgene, Italy Corporation, Milan, Italy) or vehicle (DMSO), at concentration 0.1 and 1 μM. TNF-α at 10 ng/ml (OriGene; Rockville, MD) was added to the culture medium for the last 24 h, in order to induce DC terminal maturation. At the end of culture period, both cells and CM were collected for further analysis. In some experiment, the combination of LEN and Dex (obtained by Sigma Aldrich, Milan, Italy) was tested on DC differentiation. Briefly, DCs were differentiated from BM CD14^+^ cells of MM patients, in the presence of LEN (0.1 and 1 μM) or vehicle, as reported above. At the end of culture period, cells were collected and reseeded (5 × 10^4^/ml) in fresh medium with Dex (10^−8^M) or vehicle (EtOH) for 48 h. After Dex treatment, cells were collected and analyzed for DC maturation markers. For the *ex vivo* studies, PB CD14^+^ cells were isolated from MM patients at day 0 and after one week (day 7) of LEN (25 mg/day) treatment, just before the start of the weekly treatment with Dex. Cells were then differentiated into DCs, following the above protocol, without LEN *in vitro* treatment.

DCs were also differentiated from THP-1 cell line, by adding rhIL-4 (200 ng/ml), rhGM-CSF (100 ng/ml), ionomycin (200 ng/ml) (Sigma-Aldrich, Milan, Italy) and rhTNF-α (20 ng/ml) for 72 h to the culture medium (RPMI 1640, serum depleted); then LEN or DMSO were added for the last 24 h of culture period. THP-1-derived DCs (THP1-DCs) were detached with EDTA 2 mM on ice for 2 h and cell pellets collected for further analysis.

In some experiments DCs were differentiated from BM CD14^+^ cells of MM patients, in the presence or absence of the CM (ratio 1:2 with RPMI 10% FBS, with IL-4 and GM-CSF) of hTERT-hMSCs treated with LEN or DMSO. Briefly, 1 × 10^4^ hTERT-hMSCs were seeded in T75 flasks and cultured in RPMI 10% FBS, in presence of LEN (0.1 and 1 μM) or DMSO, for 5 days. At the end of culture period, the medium was replaced with RPMI 10% FBS in order to discard LEN, and after 48 h, the CM was collected and used during DC differentiation, as previously reported. In some experiments, after 5 days of LEN treatment, hTERT-hMSC pellets were collected for immunoblotting and RT-PCR analysis.

#### Autologous mixed lymphocyte reaction and cell proliferation assay

DCs were differentiated, as previously reported, in presence of LEN or vehicle (alone or in combination with Dex), from BM of 6 MM patients, for 8 days. Then, treated cells were collected, analyzed by flow-cytometry and partly re-seeded (3 × 10^3^ cells/w) in round-bottomed 96well-plates, in RPMI 15% AB human serum. DCs were co-cultured with autologous PB CD3^+^ cells (1 × 10^4^) for 6 days. At the end of culture period, an MTT assay (Cell Counting Kit-8; Alexis, Vinci-Biochem s.r.l., Italy) was performed in order to measure T cell proliferation.

### Flow cytometry assay

After *in vitro* DC differentiation, non-adherent cells were collected and analyzed by flow cytometry for DC maturation markers. Cells from each condition were splitted in three tubes and labelled with saturating amounts of the following conjugated antibody combinations (all from BD Biosciences, San Jose, CA, USA): 1) anti-CD14-FITC/anti-CD83-PE/isotype control-PE-Cy5/isotype control-APC, 2) anti-CD14-FITC/anti-CD83-PE/anti-CD86-PE-Cy5/anti-HLA-DR-APC, 3) anti-CD14-FITC/anti-CD83-PE/anti-CD80-PE-Cy5/anti-CD209-APC. Four color, six-parameter acquisition and analysis were perfomed on a two-laser FACSCalibur instrument (BD Biosciences) using CellQuest software (BD Biosciences). Mature DCs were identified as CD14^−^CD83^+^ cells and the MFI of the maturation markers was compared between cells treated with LEN and/or Dex *vs* the relative control, for each experiment.

### Multi ELISA assay

The concentration of Interferon (IFN)-γ, IL-6, IL-8, IL-10, IL-12, IFN-γ induced protein (IP)-10, CCL2, CCL5 and TNF-α was evaluated on DC CM, collected after *in vitro* DC differentiation, by using multiplex bead-based sandwich immunoassay kits (Bio-Plex^®^ Multiplex System, Biorad, California, USA), following the manufacturer's instructions. Measurement were performed by a reader (Luminex Bio-plex system, Bio-Rad Laboratories Inc.). For TNF-α level evaluation, the obtained results were normalized for TNF-α concentration measured in the control medium (RPMI 10% FBS, with IL-4, GM-CSF and TNF-α at concentrations used during DC differentiation).

### Western blotting

Nuclear and cytosolic extracts were obtained using a commercial kit (Active Motif, Carlsbad, CA) following the manufacturer's protocol from THP1-DCs and hTERT-hMSCs, treated with LEN or DMSO. Immunoblotting was performed as previously reported [51] using the following antibodies: rabbit monoclonal anti-Aiolos (1:714) (code n. NBP2-24495, Novus Biologicals, Abingdon, UK), anti- Casein Kinase 1 (1:1.000) (code n. 2655, Cell Signaling Technology, Danvers, USA), anti-Ikaros (1:200) (code n. sc-13039, Santa Cruz Biotechnology, Dallas, USA), and mouse monoclonal anti-Cereblon (1:1.250) (code n.TA345038, OriGene, Rockville, USA), anti-IRF4 (1:400) (code n. M725929-2, DAKO, Milan, Italy); anti-p62/SQSTM1 (1:250) (Code n.MAB8028, R&D Systems, Minneapolis, MN, USA) antibodies; mouse monoclonal anti-β-actin antibody (1:5.000) (clone AC-15, code n. A5441, Sigma-Aldrich, Milan, Italy) was used as internal control. JJN3 cell line represents the positive control (CNT).

### mRNA silencing

RNAi was performed through the generation of inducible shRNA stable cell lines. hTERT-hMSC were transduced with the IPTG inducible lentiviral particles carrying CSNK1A1-specific shRNA (pLKO_IPTG_3XLacO, Sigma-Aldrich, Milan, Italy). Two independent shRNAs (TRCN0000006044, and TRCN0000006042) sequences were chosen. 3 × 10^4^ cells were infected with a multiplicity of infection (MOI) of 4, in the presence of 8 μg/ml polybrene (Sigma-Aldrich, Milan, Italy). 24 h later, the infected medium was replaced with fresh growing medium. Puromycin selection (0.5 μg/ml) was initiated 2 days after transduction. Once a cellular clone was established, to induce CK1α silencing, cells were incubated with 500 μM IPTG (Sigma-Aldrich, Milan, Italy) every 2–3 days for a total of one week. Then, fresh medium without IPTG and puromycin was added for further 48 h. At the end of culture period, cell pellets were collected and analyzed by western blotting to check CK1-α down-regulation and select the more efficient clone. CM was also collected and used for *in vitro* DC differentiation.

### RT-PCR

Total RNA was extracted from hTERT-hMSCs, after all different experimental conditions, using the RNeasy total RNA isolation kit (Qiagen; Hilden, Germany). RNA (1 μg) was reverse-transcribed with 400 U Moloney murine leukemia reverse transcriptase (Applied Biosystems, Life Technologies, Carlsbad, CA, USA) in accordance with the manufacturer's protocol. Real Time PCR was performed by adding complementary DNA to a universal Light Cycler 480 Probes Master and RealTime ready Catalog Assay (Roche Diagnostics, Mannheim, Germany) for the following genes: *IDO1* (Assay ID:103804), *IL6* (Assay ID: 144013), *CCL5* (Assay ID: 113395), *PTGS2* (Assay ID: 102471), *TGB1* (Assay ID: 101210), and *GAPDH* (Assay ID: 102052). The expression of selected genes was checked by Real Time PCR by Light Cycler 480 (Roche Diagnostics, Mannheim, Germany). To normalize the differences in RNA quality and reverse transcription efficiency, we applied the comparative Ct method using the endogenous reference gene *GAPDH*.

### Statistical analysis

Data were expressed as mean ± SEM or median values. Paired Student's *t*-test was used to analyze flow cytometry data of *in vitro* DC differentiation from BM and PB of MM patients and for the *ex vivo* studies. Non-parametric Friedman test, Wilcoxon test and Mann-Whitney test were used for the other experiments with a lower number of samples. Results were considered significant at *p* < 0.05. GraphPad Prism 6.1^™^ (GraphPad Software Inc., La Jolla, CA, USA) was used for all the statistical analyses.

## SUPPLEMENTARY FIGURES



## Abbreviations

IMiDs^®^: Immunomodulatory drugs
LEN: Lenalidomide
POM: Pomalidomide
MM: Multiple myeloma
T reg: T regulatory cell
ADCC: Antibody-dependent cell-mediated citotoxicity
NK: Natural killer
CRBN: Cereblon
CRL: Cullin-RING E3 ubiquitin ligase
CK1-α: Casein kinase 1 alpha
IL: interleukin
MDS: myelodysplastic
del(5q): deletion of chromosome 5q
DCs: dendritic cells
PB: peripheral blood
aGvHD: acute graft versus host disease
MSCs: mesenchymal stromal cells
APCs: antigen presenting cells
Mo-DCs: monocytes derived dendritic cells
BM: bone marrow
SEM: standard error of the mean
Dex: Dexamethasone
CCL: CC chemokine ligand
TNF-α: tumor necrosis factor alpha
MLR: Mixed Lymphocyte Reaction
hTERT-hMSCs: human telomerase reverse transcriptase transduced mesenchymal stromal cells
CM: conditioned medium
RT-PCR: Real Time PCR
PTGS2: prostaglandine 2
IDO1: indoleamine 2,3-dioxygenase1
TGFB1: Transforming growth factor beta 1
SMM: Smoldering MM
MNCs: Mononuclear cells
rh: recombinant human
GM-CSF: granulocyte macrophage colony-stimulating factor
HMCL: Human myeloma cell line
mAb: Monoclonal antibody
ISS: International staging system
IFN-γ: Interferon gamma
THP1-DCs: THP-1 derived DCs
IP10: IFN-γ induced protein 10
