# Supplementary material for: Lenalidomide increases human dendritic cell maturation in multiple myeloma patients targeting monocyte differentiation and modulating mesenchymal stromal cell inhibitory properties
Source: Oncotarget. 2017 May 23;8(32):53053–67. doi: 10.18632/oncotarget.18085 (PMC5581092; doi:10.18632/oncotarget.18085)
Supplement: Supplementary file 1 [file oncotarget-08-53053-s001.pdf]

Lenalidomide increases human dendritic cell maturation in multiple myeloma patients targeting monocyte differentiation and modulating mesenchymal stromal cell inhibitory properties

Supplementary Materials

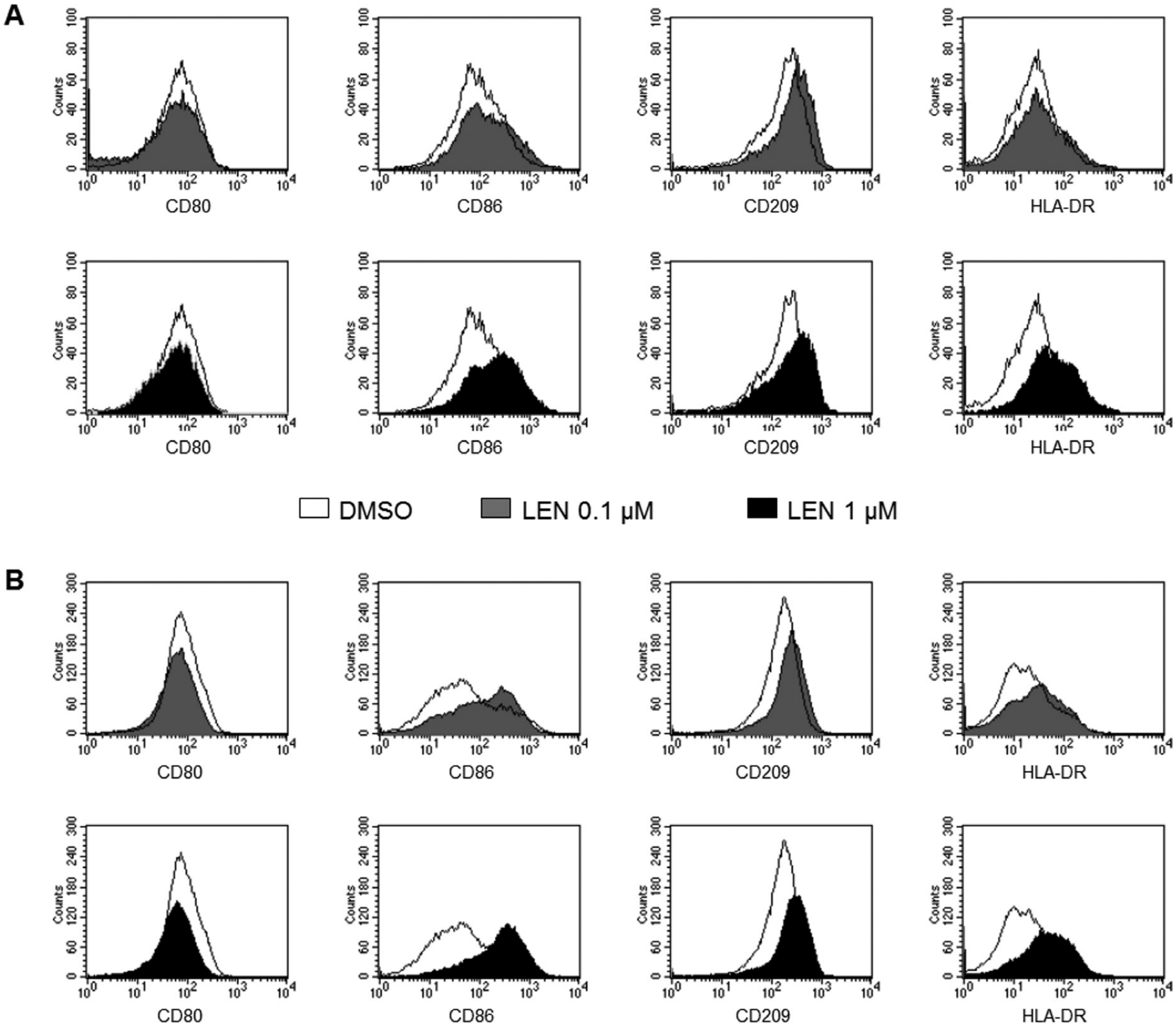

Supplementary Figure 1: LEN treatment increased DC maturation markers in both BM and PB of MM patients. DCs were differentiated from BM and PB CD14^{+} cells of MM patients, cultured in RPMI 10% FBS with IL-4 and GM-CSF, for 8 days, in presence of LEN (0.1 and 1 µM) or DMSO. TNF-α was added in the last 24 h of differentiation period. Non-adherent cells were collected and analysed by flow-cytometry for DC maturation markers. Representative flow-cytometry histograms from BM (**A**) and PB (**B**) of 2 MM patients were reported. Empty histograms: DMSO, gray histograms: LEN 0.1 µM, black histograms: LEN 1 µM.

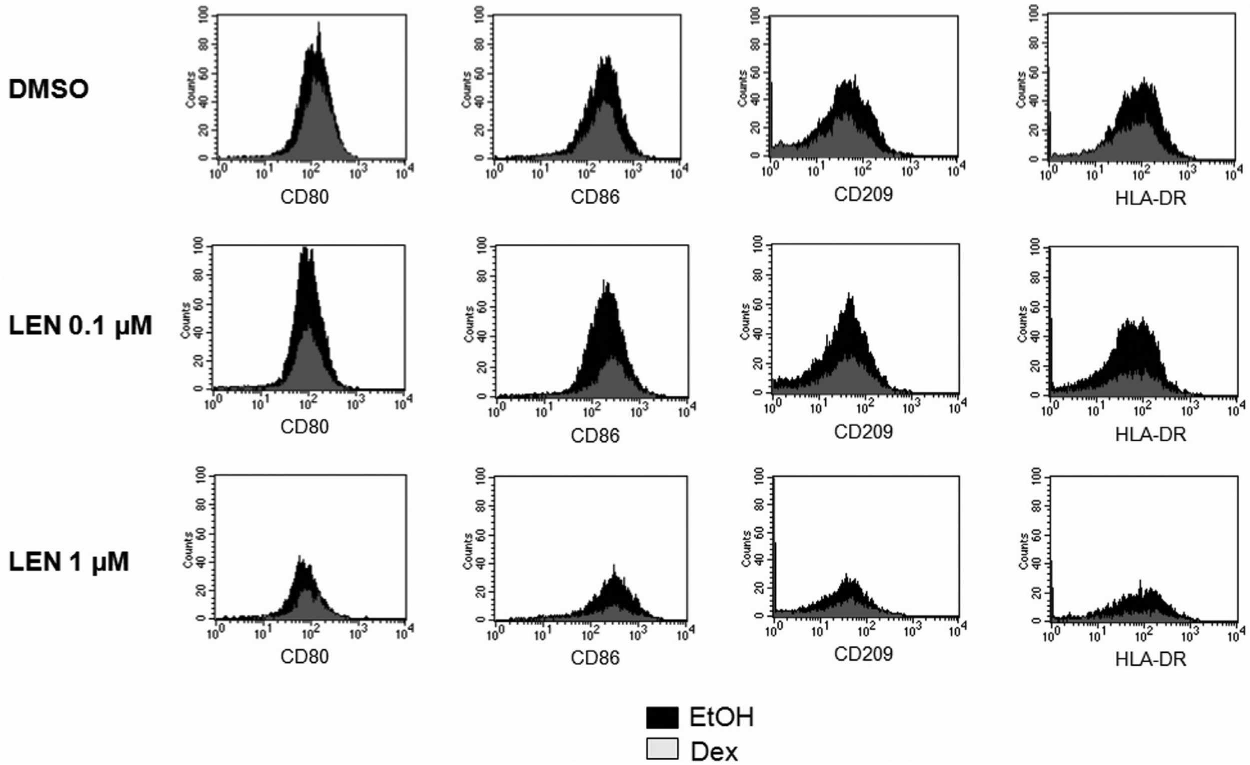

Supplementary Figure 2: LEN effect on DC maturation markers was abrogated by the combination with Dexamethasone. DCs were differentiated from BM CD14^{+} cells of MM patients, in the presence of LEN (0.1 and 1 μM) or DMSO. At the end of culture period, cells were collected and reseeded (5×10^{4}/ml) in fresh medium with Dex (10^{-8}M) or vehicle (EtOH) for 48 h. After Dex treatment, cells were collected and analyzed for DC maturation markers. Representative flow-cytometry histogram from one MM patient. Black histograms: DMSO or LEN + EtOH (vehicle). Gray histograms: DMSO or LEN + Dex 10^{-8} M.

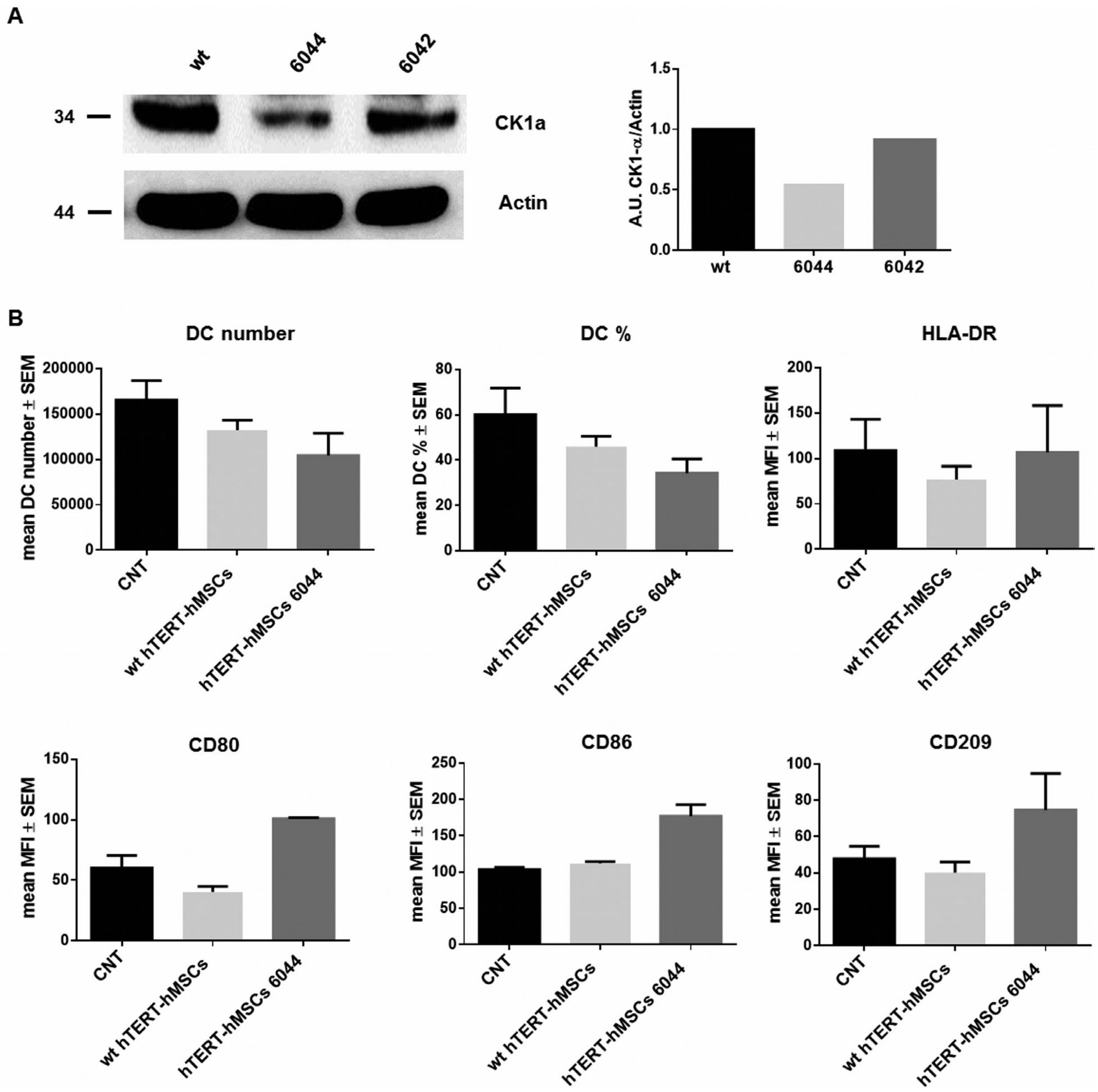

Supplementary Figure 3: LEN reverted the immunosuppressive properties of hTERT-hMSCs through the down-regulation of CK1-α. CK1-α down-regulation was obtained by hTERT-hMSCs transduction with the IPTG inducible lentiviral particles carrying CSNK1A1-specific shRNA (clone 6044 and 6042). At the end of culture period, cell pellets were collected and analyzed by western blotting to check CK1-α down-regulation and select the efficient clone. Wilde type (wt) hTERT-hMSCs were used as control. β-actin was used as internal control for western blotting. (**A**) DCs were differentiated from BM $CD14^{+}$ cells of MM patients, in the presence or absence (CNT) of the CM (ratio 1:2 with RPMI 1640 10% FBS, with IL-4 and GM-CSF) of hTERT-hMSCs transduced with the more efficient clone 6044. At the end of culture period, cells were collected and analyzed for DC maturation markers, by flow-cytometry. Graph bar represent the mean ± SEM of DC number and % and the mean MFI of DC maturation markers of two independent experiments (**B**).

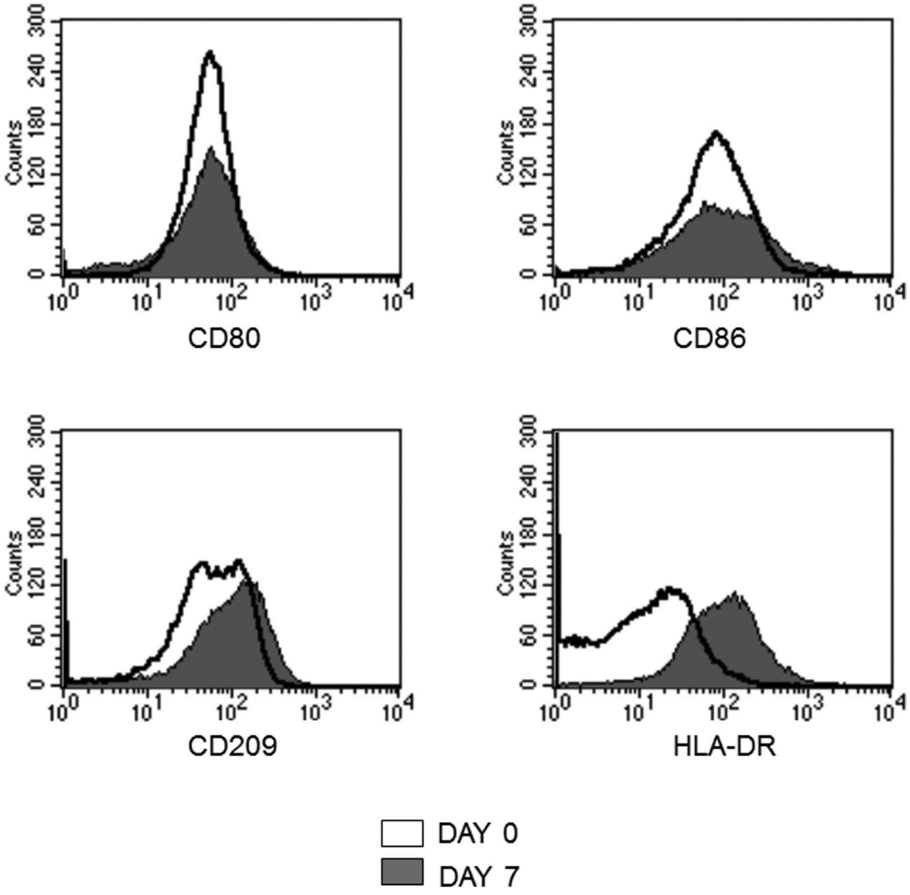

Supplementary Figure 4: *In vivo* LEN treatment of MM patients increased *in vitro* DC differentiation. DCs were *in vitro* differentiated from PB CD14^{+} cells of MM patients at DAY 0 and after one week (DAY 7) of LEN 25 mg/day treatment. Cells were cultured in RPMI 10% FBS with IL-4 and GM-CSF for 8 days and TNF-α was added for the last 24 h. At the end of culture period, cells were collected and analyzed for DC maturation markers, by flow-cytometry. Representative flow-cytometry histograms from one MM patients were reported. Empty histograms: DAY 0, gray histograms: DAY 7.
